# Directed evolution of a fungal β-glucosidase in *Saccharomyces cerevisiae*

**DOI:** 10.1186/s13068-016-0470-9

**Published:** 2016-03-03

**Authors:** Kane Larue, Mindy Melgar, Vincent J. J. Martin

**Affiliations:** Department of Biology, Centre for Structural and Functional Genomics, Concordia University, 7141 Sherbrooke West, Montreal, QC H4B 1R6 Canada

**Keywords:** Biofuels, Consolidated bioprocessing, β-glucosidase, Cellulase, Inhibition, Directed evolution

## Abstract

**Background:**

β-glucosidases (BGLs) catalyze the hydrolysis of soluble cellodextrins to glucose and are a critical component of cellulase systems. In order to engineer *Saccharomyces cerevisiae* for the production of ethanol from cellulosic biomass, a BGL tailored to industrial bioconversions is needed.

**Results:**

We applied a directed evolution strategy to a glycosyl hydrolase family 3 (GH3) BGL from *Aspergillus niger* (BGL1) by expressing a library of mutated *bgl1* genes in *S. cerevisiae* and used a two-step functional screen to identify improved enzymes. Twelve BGL variants that supported growth of *S. cerevisiae* on cellobiose and showed increased activity on the synthetic substrate *p*-nitrophenyl-β-D-glucopyranoside were identified and characterized. By performing kinetic experiments, we found that a Tyr → Cys substitution at position 305 of BGL1 dramatically reduced transglycosidation activity that causes inhibition of the hydrolytic reaction at high substrate concentrations. Targeted mutagenesis demonstrated that the position 305 residue is critical in GH3 BGLs and likely determines the extent to which transglycosidation reactions occur. We also found that a substitution at Gln^140^ reduced the inhibitory effect of glucose and could be combined with the Y305C substitution to produce a BGL with decreased sensitivity to both the product and substrate. Using the crystal structure of a GH3 BGL from *A. aculeatus*, we mapped a group of beneficial mutations to the β/α domain of the molecule and postulate that this region modulates activity through subunit interactions. Six BGL variants were identified with substitutions in the MFα pre-sequence that was used to mediate secretion of the protein. Substitutions at Pro^21^ or Val^22^ of the MFα pre-sequence could produce up to a twofold increase in supernatant hydrolase activity and provides evidence that expression and/or secretion was an additional factor limiting hydrolytic activity.

**Conclusions:**

Using directed evolution on BGL1, we identified a key residue that controls hydrolytic and transglycosidation reactions in GH3 BGLs. We also found that several beneficial mutations could be combined and increased the hydrolytic activity for both synthetic and natural substrates.

**Electronic supplementary material:**

The online version of this article (doi:10.1186/s13068-016-0470-9) contains supplementary material, which is available to authorized users.

## Background

Cellulosic biorefineries offer the potential to produce fuels and other chemicals from renewable substrates [1]. While advances have been made towards economically viable bioconversion processes, the efficiency and cost of saccharolytic enzymes are obstacles that challenge the feasibility of the large-scale production of fermentable sugars from cellulosic plant biomass. A promising strategy is to engineer an organism that expresses a functional cellulase system that simultaneously hydrolyses biomass while fermenting hydrolysate [2–4]. Consolidated bioprocessing (CBP) would eliminate the costs of a dedicated saccharification process but depends on the development of a fermentation strain expressing a cellulase system capable of efficiently hydrolyzing cellulosic biomass at high solid loadings.

Saccharolytic enzymes from cellulolytic microbes are well characterized and are excellent candidates for industrial applications [5]. In the minimal model of cellulose hydrolysis, cellobiose and other soluble cellodextrins are released through the synergistic activities of endoglucanases (EGLs; EC 3.2.1.4) and cellobiohydrolases (CBHs; 3.2.1.91). β-glucosidases (BGLs; 3.2.1.21) are a third and critical component of natural and engineered cellulase systems. Their role is twofold: The hydrolysis of soluble cellodextrins by BGLs produce a fermentable sugar, glucose, while removing hydrolysate intermediates that act as inhibitors toward EGLs and CBHs [6]. Unfortunately, most characterized microbial BGLs perform best at low substrate concentrations, with *K*_*m*_ values between 1–3 mM for cellobiose [7–9]. BGLs are also inhibited by glucose [6, 10], with reported *K*_*i*_ values between 1–10 mM [11, 12]. Glucose-tolerant BGLs have been identified [13–16], but the mechanism of glucose tolerance is not understood. In addition to glucose sensitivity, inhibition of hydrolase activity is often observed at substrate concentrations above *K*_*m*_, where transglycosidation reactions occur [7]. Inhibition of BGL hydrolytic activity by product and substrate contribute to limiting the efficiency of cellulosic bioconversion processes because the saccharification of biomass by EGLs and CBHs is dependent on the hydrolysis of soluble cellodextrins to glucose [17].

The yeast *Saccharomyces cerevisiae* is well-suited as a CBP platform microorganism because it naturally ferments hexose sugars to ethanol at high yields. However, *S. cerevisiae* is non-cellulolytic and a CBP strain designed to secrete cellulases is needed. Two challenges remain in the development of a strain of *S. cerevisiae* with cellulolytic activity: First, an efficient set of saccharolytic enzymes must be optimized for the hydrolysis of pre-treated cellulosic biomass. Second, recombinant cellulases must be secreted at sufficient levels such that the production of sugars will support ethanol fermentation at suitable rates and yields. The heterologous expression of saccharolytic enzymes in *S. cerevisiae* and other hosts is well documented [18–23]. BGLs secreted from *S. cerevisiae* enable growth on cellobiose [24–27], and the co-expression of BGLs with other cellulases enable growth on pure cellulose or pre-treated cellulosic feedstocks [20, 21, 28, 29], but low ethanol yields have been reported from cellobiose (<20 g/L [19]) and amorphous cellulose (<7 g/L [30]). Experiments using *Trichoderma reesei* have shown that the addition of exogenous BGL to a natural cellulase system increases the efficiency of cellulose hydrolysis [31, 32]. Since *S. cerevisiae* secretes saccharolytic enzymes well below titers reported for other proteins [33], increasing BGL titers would likely improve fermentation yields and is therefore a key objective for CBP strain development.

BGL inhibition and low expression are compounding obstacles to the development of CBP using *S. cerevisiae.* With the goal of identifying the best BGL for use in industrial processes, many studies have been based on the discovery and comparative characterization of enzymes from cellulolytic organisms [11, 22, 34, 35]. Attempts have also been made at improving natural BGLs by reducing inhibition through rational design [36], the construction of gene fusions [37, 38], increasing expression via codon optimization [25], and using alternative secretion or cell anchoring strategies [25, 39, 40]. Structural data are also available for several BGLs, with representative structures from GH1 and GH3 proteins [41–43]. Despite the scope of information available, a BGL tailored to CBP is yet to be discovered or engineered [44].

Directed evolution is a proven tool for protein engineering. Using an artificial selection process to screen mutagenized proteins, enzyme function can be altered or optimized to meet specific needs [45, 46]. The use of directed evolution to modify cellulases for industrial applications has been demonstrated on EGLs [47–49] and BGLs [50–55], where thermal stability, activity at non-natural temperatures, and pH optimum are most often targeted for improvement. With the goal of building a CBP strain, we applied a directed evolution strategy based on a two-step functional screen to identify enzyme variants of a GH3 BGL from *Aspergillus niger* using *S. cerevisiae* as a host. This strategy offers the potential to remodel a BGL for increased hydrolysis activity while improving heterologous expression in an efficient fermentation strain.

## Results

### Directed evolution of BGL1

**Fig. 1:**
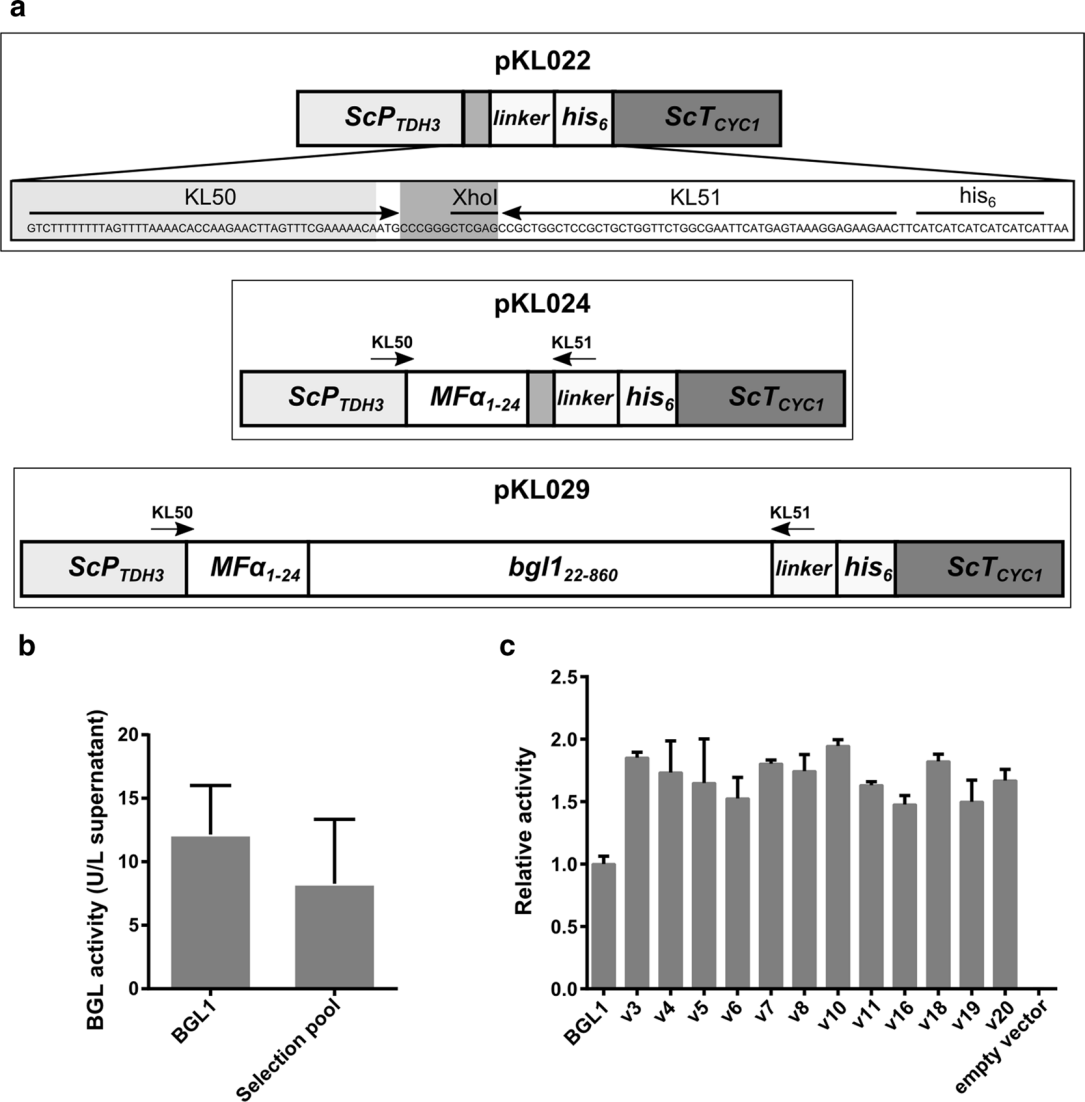
Directed evolution of BGL1. **a** Schematic of the assembly and expression cassettes from pKL022, pKL024, and pKL029. **b** Activities of a wild-type population and selection pool from endpoint assays. **c** Activities of improved BGLs determined using time course assays. Relative enzyme activities were normalized to cell density. *Error bars* represent mean ±95 % confidence interval of triplicate experiments

We targeted the β-glucosidase from *A. niger* (BGL1) using directed evolution to adapt it towards heterologous expression *S. cerevisiae*. We built a fusion protein by replacing the native BGL1 signal sequence with the *S. cerevisiae* mating pheromone α factor (MFα) pre-sequence to direct secretion of the protein into culture media. Our strategy utilized the native homologous DNA recombination machinery in *S. cerevisiae* to assemble a library of mutagenized *bgl1* genes, followed by a two-step selection to identify improved mutants. Because wild-type *S. cerevisiae* lacks β-glucosidase activity (Fig. 1c; empty vector), growth on cellobiose was used as the primary selection method. We then employed an endpoint assay using pNPG as a substrate for quantitative measurement of β-glucosidase activity. To generate sequence diversity, the *bgl1* gene was mutagenized using error-prone PCR and transformants were cultured as a mutant pool in liquid media to maximize the library size. We determined that the pooled library contained approximately 1.6–2 × 10^7^ recombinant mutants by growing small quantities (0.0001–0.1 %) of the transformation mixture on solid media immediately after electroporation. Approximately 3 × 10^5^ variants (1.5–2 % of the total library) were subsequently screened on solid media containing cellobiose as the sole carbon source. Ninety-five percent of the mutant *bgl1* library clones did not grow on cellobiose, showing that most mutations were deleterious. Colonies from the mutant pool varied in size whereas those expressing the experimental wild-type enzyme showed no observable variation. This allowed colony size to be used as a semi-quantitative screen for BGL activity, as has been previously reported [56–58]. The BGL activity secreted from cells originating from the largest colonies was measured using *p*-nitrophenyl-β-D-glucopyranoside (pNPG) as the substrate and compared to cultures expressing the BGL1 protein. The mean activity of the mutant pool decreased while increasing in variability (Fig. 1b), following the predicted trend for a library of mutagenized enzymes [59]. Of 1380 variants screened, twelve mutants met the threshold cutoff for selection of wild-type + 2SD (Fig. 1c).

Sequencing showed that the mutant *bgl1* genes each had 3–9 nucleotide substitutions (Table 1), corresponding to a mutation rate of 1.6 ± 0.4 bp per kb. Certain mutations occurred in multiple variants (65T > A: v7 and v18; 65T > C: v4 and v19; 428A > T: v5 and v20 and 1707C > T: v4 and v6). Six variants had mutations encoding amino acid substitutions immediately following the predicted MFα signal peptide cleavage site at Pro^21^ or Val^22^. These two amino acids were targeted by four substitutions (P21T, P21S, V22D, and V22A). Using the prevalence of substitutions at positions 21 and 22 of the MFα pre-sequence, we divided the evolved BGL1s into two groups based on the presence or absence of substitutions at Pro^21^ and Val^22^. For the group of BGLs with signal peptide substitutions, we constructed genes with single mutations encoding the P21T, P21S, V22D, and V22A. For the remaining BGL1 variants, we constructed genes to test all of the mutations individually. We chose to include silent mutations in our experiments to investigate if codon optimization had occurred. All of the substitutions tested at positions 21 and 22 produced increases in activity similar to those of their parental evolved enzymes containing two or more mutations (Table 1), indicating that mutations in the MFα pre-sequence increased protein secretion to the culture media. None of the silent mutations tested produced observable increases in activity. We were able to establish a relationship between the increase in activity observed for several of the evolved BGLs and a single amino acid substitution (v3: Y305C; v5 and v20: Q140L; v10: A480V), but none of the mutations tested individually could account for the activities of v6 and v16 (Table 1). The K494Q and N557D substitutions did produce significant and reproducible improvements (117 and 119 %, respectively), suggesting a cumulative effect that contributed to the 147 % increase in activity observed for v16. We chose to characterize the A480V substitution (1448C > T) in the genetic context of v10 since the other mutations present in the gene (1180C > T and 1506T > A) were silent and did not produce any improvements when tested alone.

**Table 1:** Characterization of improved BGLs

Variant	Mutation	Amino acid	Substitution	Relative activity
v3	923A > G	305	Tyr → Cys	1.68 ± 0.02
	1557A > T	516	–	1.05 ± 0.01
	1934A > T	642	Glu → Val	0.80 ± 0.03
v4^a^	65T > C	22	Val → Ala	1.57 ± 0.06
	174A > T	55	–	
	465C > T	152	–	
	1707C > T	566	–	0.98 ± 0.03
v5	428A > T	140	Gln → Leu	1.56 ± 0.05
	2067A > T	686	–	1.03 ± 0.02
	2103T > A	698	–	1.07 ± 0.03
v6	917T > C	303	Val → Ala	1.09 ± 0.04
	1707C > T	566	–	0.98 ± 0.03
	1818T > C	603	–	1.05 ± 0.04
v7^a^	65T > A	22	Val → Asp	2.03 ± 0.08
	297T > C	96	–	
	643A > G	212	Ile-Val	
	892T > C	295	–	
	1814A > G	602	Lys → Arg	
	2019T > C	670	–	
	2079T > A	690	–	
v8^a^	25G > A	9	Ala → Thr	
	60T > A	20	–	
	61C > A	21	Pro → Thr	1.77 ± 0.02
	93T > C	28	–	
	462T > C	151	–	
	981C > T	324	–	
	1158C > T	383	–	
	1953C > T	648	–	
	2389A > C	794	Lys → Gln	
v10	1180T > C	391	–	0.90 ± 0.02
	1448C > T	480	Ala → Val	Not determined
	1506T > A	499	–	0.88 ± 0.05
v11^a^	61C > T	21	Pro → Ser	1.93 ± 0.02
	366T > C	119	–	
	954C > T	315	–	
	1773C > T	588	–	
v16	681T > C	224	–	0.86 ± 0.01
	1489A > C	494	Lys → Gln	1.17 ± 0.06
	1678A > G	557	Asn → Asp	1.19 ± 0.06
v18^a^	65T > A	22	Val → Asp	2.03 ± 0.08
	1983C > T	658	–	
v19^a^	65T > C	22	Val → Ala	1.57 ± 0.06
	219G > A	70	–	
	1275A > G	422	–	
	2367T > G	786	–	
	2441C > T	811	Thr → Met	
v20	428A > T	140	Gln → Leu	1.56 ± 0.05
	1925A > T	639	Lys → Met	0.73 ± 0.04
	2349T > A	780	–	0.94 ± 0.01

^a^Variants for which only signal sequence mutations at residues 21 or 22 were investigated

### Kinetic characterization of BGL mutants using pNPG

**Fig. 2:**
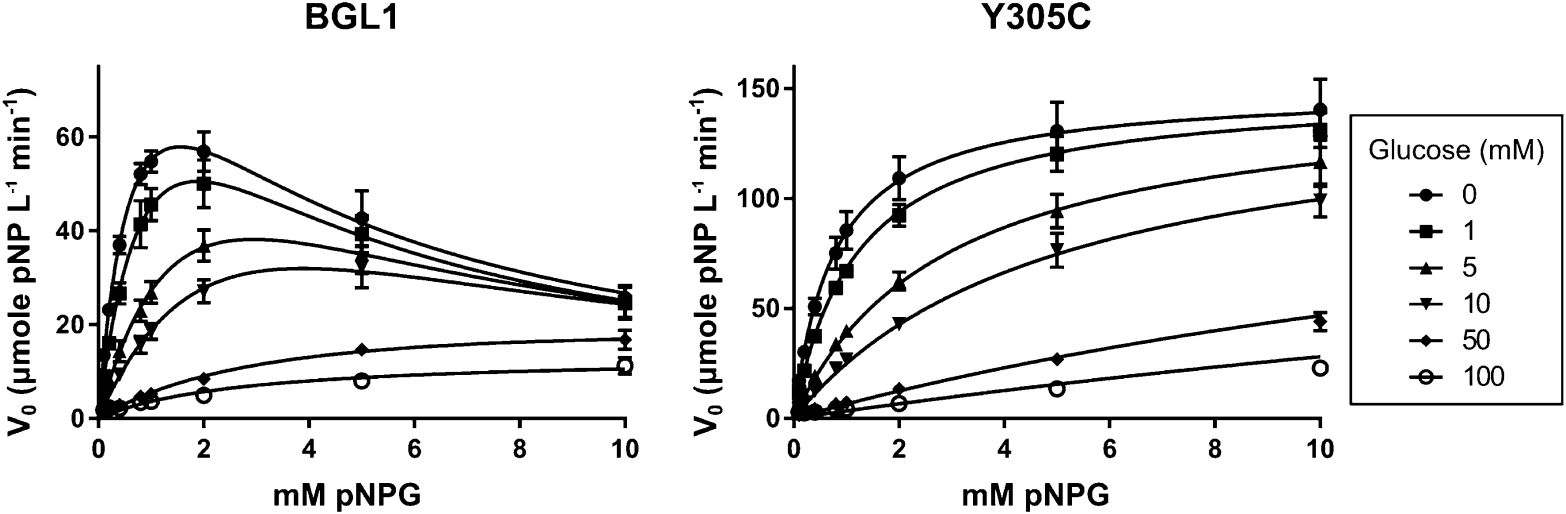
Representative V_*0*_ versus substrate concentration plots for the production of pNP at a range of pNPG concentrations in the presence of inhibitor

We further characterized the substitutions producing the greatest improvements with kinetic experiments. Initial reaction velocities were measured for each BGL variant at different substrate concentrations (0.1–10 mM pNPG) and at different inhibitor concentrations (0–100 mM glucose) (Table 2). Wild-type and all single substitution variants, except Y305C, were fitted to a substrate inhibition model (Fig. 2). Y305C showed no inhibition at high substrate concentrations and the reaction velocities were fitted to the Michaelis–Menten equation using a competitive inhibition model (Fig. 2). Most of the variants showed an increase in _*app*_*V*_*max*_ similar to the relative activities reported from activity assays at 2 mM pNPG. No other significant differences were observed in reaction kinetics between BGL1 and the mutant enzymes. Based on the change in kinetic profile caused by the Cys substitution at position 305, we used Y305C as a background to further explore the contributions of the V22D, Q140L, and A480V substitutions. We engineered V22D/Y305C, Q140L/Y305C, Y305C/A480V double-substitution variants and a quadruple DLCV (V22D/Q140L/Y305C/A480V) variant. Reaction velocities for all of the combinatorial variants were modeled using the Michaelis–Menten equation and kinetic parameters were determined (Table 3). As expected, the addition of V22D, Q140L, and A480V to Y305C increased the _*app*_*V*_*max*_ for each enzyme. _*app*_*K*_*m*_ and _*app*_*K*_*i* glucose_ for V22D/Y305C and Y305C/A480V were similar to Y305C, while the inhibitory effect of glucose was slightly reduced for Q140L/Y305C and DLCV.

**Table 2:** Kinetic parameters of BGL1 and evolved variants for synthetic substrate

	_*app*_ *K*_*m*_ (mM)	_*app*_ *V*_*max*_ (μmole L^−1^ min^−1^)	_*app*_ *K*_*i* glucose_ (mM)	_*app*_ *K*_*i* pNPG_ (mM)
BGL1	0.82 ± 0.12	118.5 ± 10.6	–	2.98 ± 0.46
V22D	0.93 ± 0.23	210.5 ± 31.6	–	2.98 ± 0.75
Q140L	1.09 ± 0.06	249.6 ± 9.2	–	3.41 ± 0.21
Y305C	0.77 ± 0.03	149.7 ± 1.7	1.81 ± 0.08	–
A480V	0.98 ± 0.25	217.7 ± 34.3	–	3.26 ± 0.87
K494Q	0.89 ± 0.12	141.5 ± 11.1	–	3.06 ± 0.41
N557D	0.88 ± 0.09	186.2 ± 11.0	–	3.16 ± 0.32

**Table 3:** Kinetic parameters of Y305C substituted variants for synthetic substrate

	_*app*_ *K*_*m*_ (mM)	_*app*_ *V*_*max*_ (μmole mg^−1^ min^−1^)	_*app*_ *K*_*i* glucose_ (mM)
Y305C	0.93 ± 0.04	71.0 ± 0.9	1.92 ± 0.10
V22D/Y305C	1.06 ± 0.04	127.4 ± 1.3	2.22 ± 0.09
Q140L/Y305C	1.32 ± 0.05	111.1 ± 1.4	3.18 ± 0.15
Y305C/A480V	1.06 ± 0.05	124.3 ± 1.8	2.22 ± 0.12
DLCV	1.69 ± 0.08	221.3 ± 3.6	3.37 ± 0.18

### Structural mapping of BGL substitutions

**Fig. 3:**
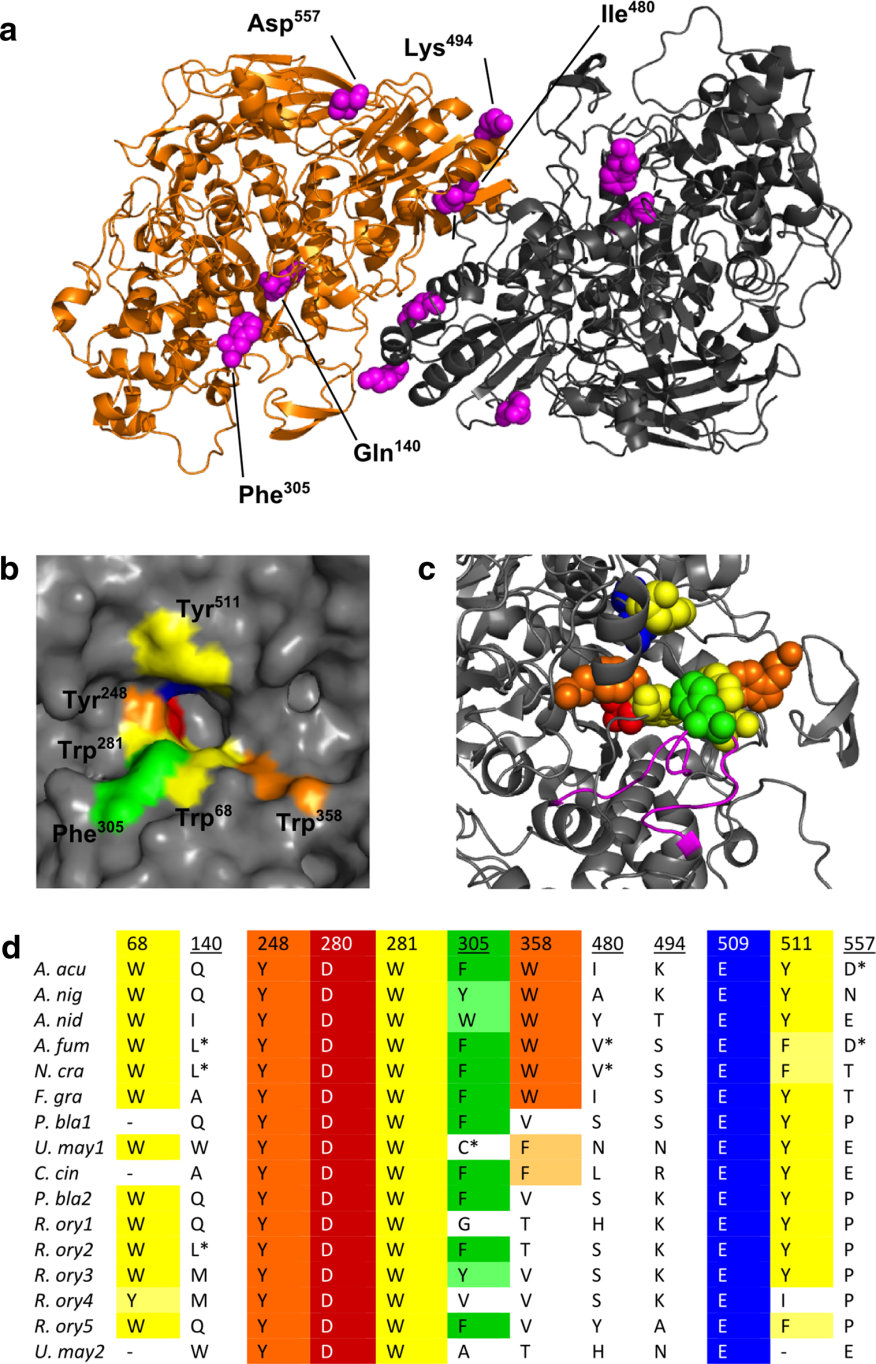
Structure/function analysis of beneficial mutations in GH3 BGLs. **a** Molecular mapping of mutations using the *A. aculeatus* BGL1 crystal structure (PDB 4IIB). Chain A *orange*; Chain B *gray*. Substitutions identified by mutagenesis and functional selection are shown in *magenta* and labeled on Chain A. **b** Residues contributing to the substrate-binding pocket of *A. aculeatus* BGL1. **c** Gly^294^–Gly^313^ residues (*magenta*) coordinate Phe^305^ (*green*) in the +1 subsite. **d** Alignments of GH3 residues. Residues identified by directed evolution are underlined. *Asterisks* (*) indicate beneficial substitutions found in nature. *A. acu Aspergillus aculeatus* BGL1*; A. nig Aspergillus niger* BGL1*; A. nid Apergillus nidulans; A. fum Aspergillus fumigatus; N. cra Neurospora crassa; F. gra Fusarium graminearum; P. bla Phycomyces blaskesleeanus; U. may Ustilago maydis; C. cin Coprinopsis cinerea; R. ory Rhizopus oryzae.* *Color coding* corresponds between (**b**), (**c**), and (**d**). Multiple sequence alignments were performed using Clustal Omega [73]. Structural analyses were performed using PyMOL

We used the three-dimensional structure of *Aspergillus aculeatus* BGL1 (AaBGL1) [41] to map the hypothetical position of the substitutions identified through directed evolution (Fig. 3a). Multiple sequence alignments suggest that BGL1 and other closely related fungal GH3 BGLs would adopt a three-dimensional structure similar to AaBGL1. BGL1 shares 83 % sequence identity with AaBGL1, and residues forming the active site are well conserved (Fig. 3b, d). The Q140L substitution maps to the triosephosphate isomerase domain (AaBGL1 Leu^19^–Ser^356^) and is 9.1 Å from the closest substrate-binding residue (AaBGL1 Trp^280^) and is approximately 10 Å from the β/α sandwich domain. The A480V, K494Q, and N557D substitutions are located on the β/α sandwich domain (AaBGL1 Gln^385^–Gly^588^) that contributes two active site residues (AaBGL1 Glu^509^ and Tyr^511^) and also forms the major interface between subunits. The A480V (AaBGL1 Ile^480^) substitution is located on the surface of each subunit buried between the dimer interface. Both the K494Q and the N557D mutations (AaBGL1 Lys^494^ and Asp^557^) are located on the surface of the molecule. Lys^494^ is also proximal to the dimer interface, though it does not contact the opposite subunit directly. The Y305C substitution maps to a short loop (AaBGL1 Gly^294^–Gly^313^) that inserts directly into the +1 subsite of AaBGL1 along with Trp^68^ and Tyr^511^. The structure of AaBGL1 in complex with thiocellobiose shows the contribution of Phe^305^ in the substrate-binding pocket, where the ligand docks in a narrow cleft between the three +1 subsite residues. Since the Cys functional group is less bulky than Tyr, we assume that the +1 subsite of the Y305C variant is more open and has a lower affinity for substrate.

### Targeted mutagenesis

To determine if residue 305 controls hydrolytic versus transglycosidase activities in other homologs, we investigated the relative location in other GH3 BGLs (Fig. 3d). Phe is the most common residue at position 305 (9/16 sequences) and has a similar aromatic functional group as the Tyr residue from BGL1. The position was variable in the remaining seven homologs and included a Cys residue in the BGL from *Ustilago maydis*, which would suggest that its active site is similar to the Y305C variant. We sought to further test the functional significance of this position by constructing Y305F, Y305W, Y305G, Y305V, and Y305A BGL1 variants. The Y305F BGL1 variant had a similar kinetic profile to the wild-type enzyme, demonstrating inhibition at high substrate concentrations (Table 4). However, the Y305F variant had a lower _*app*_*K*_*m*_ and higher _*app*_*K*_*i* pNPG_ than BGL1, suggesting a slight change in affinity for acceptor at the +1 subsite. Substituting Tyr^305^ with Ala, Val, or Gly produced kinetics similar to the Y305C mutant, demonstrating saturation at high substrate concentrations. Sequence alignments identified a Trp residue in the BGL from *Aspergillus nidulans*, but the Y305W variant was non-functional and suggests that the bulky functional group blocked the substrate-binding pocket. Closer inspection of sequence alignments shows that the loop coordinating position 305 residue (Gly^294^–Gly^313^) (Fig. 3c) contains a short variable region (residues 303–309) between two highly conserved sequences (Gly^294^–Asp^302^ and Ser^310^–Gly^313^). The corresponding variable region from the *A. nidulans* BGL (^438^GLHWADG^444^) includes an additional residue. It is likely that the Trp residue does not occupy the +1 subsite in the *A. nidulans* homolog and either a His or Ala is present in the substrate-binding pocket of the enzyme. Alternatively, the additional residue in the *A. nidulans* sequence could change the orientation of the Trp residue in the +1 subsite such that the substrate-binding pocket is not blocked.

**Table 4:** Kinetic parameters of Tyr^305^ variants for synthetic substrate

	_*app*_ *K*_*m*_ (mM)	_*app*_ *V*_*max*_ (μmole L^−1^ min^−1^)	_*app*_ *K*_*i* pNPG_ (mM)
Y305F	0.46 ± 0.04	126.5 ± 5.4	4.78 ± 0.44
Y305W^a^	NA	NA	NA
Y305G	1.16 ± 0.06	239.7 ± 3.9	–
Y305A	1.55 ± 0.06	238.2 ± 4.1	–
Y305 V	1.14 ± 0.08	233.9 ± 5.6	–

^a^Inactive under all tested conditions

### Enzyme kinetics using natural substrate

**Fig. 4:**
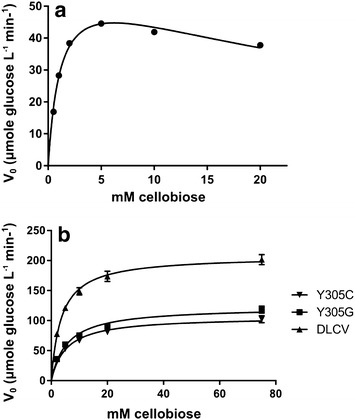
Reaction rates for the production of glucose at a range of cellobiose concentrations. **a** BGL1; **b** engineered variants

Since selection in our screening strategy was based on the hydrolysis of cellobiose, we also investigated reaction kinetics using the natural substrate for wild-type and mutant enzymes. The production of glucose was measured at different cellobiose concentrations for BGL1, Y305C, Y305G, and DLCV enzymes and kinetic parameters were determined (Table 5). The results were consistent with experiments using synthetic substrate, where the wild-type enzyme showed inhibition at high cellobiose concentrations (Fig. 4a). Y305C, Y305G, and DLCV were modeled using Michaelis–Menten kinetics, showing saturation and high substrate concentrations (Fig. 4b).

**Table 5:** Kinetic parameters of BGLs for natural substrate

	_*app*_ *K*_*m*_ (mM)	_*app*_ *V*_*max*_ (μmole L^−1^ min^−1^)	_*app*_ *K*_*i* cellobiose_ (mM)
BGL1	1.2 ± 0.1	61.9 ± 2.5	32.4 ± 4.5
Y305C	5.3 ± 0.6	106.4 ± 3.4	–
Y305G	5.6 ± 0.7	122.4 ± 4.3	–
DLCV	3.6 ± 0.3	207.5 ± 4.3	–

### TLC analysis of reaction products

**Fig. 5:**
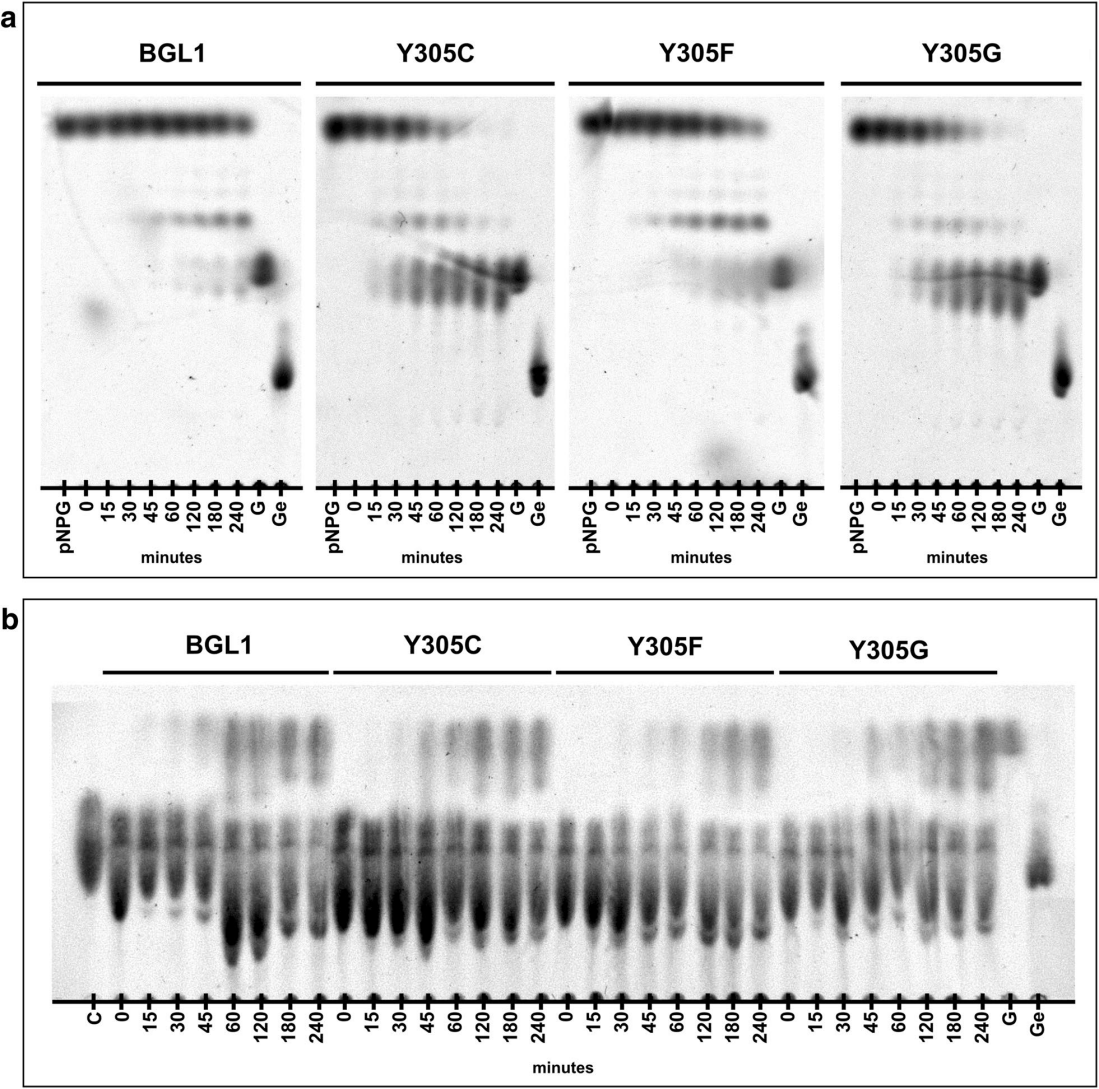
Thin-layer chromatography of BGL reactions using (**a**) 40 mM pNPG and (**b**) 50 mM cellobiose. Standards (1 μl) were 40 mM pNPG, 50 mM cellobiose (C), 50 mM glucose (G), and 25 mM gentiobiose (Ge)

Transglycosidation reactions catalyzed by BGLs with natural and synthetic acceptors are thought to be the cause of inhibition at high substrate concentrations [7–9, 12, 60–64]. Since kinetic experiments suggested that the transglycosidation activity of BGL1 was lost by substituting Tyr^305^ with either Cys, Gly, Ala, or Val residues, we performed thin-layer chromatography (TLC) analysis of reactions at high substrate concentrations (Fig. 5a, b). Kinetic experiments showed that BGL1 is strongly inhibited by synthetic substrate, where *V*_*0*_ was reduced by approximately 50 % at 10 mM pNPG. Analysis of reaction products confirmed that BGL1 and the Y305F variant were inhibited at 40 mM pNPG, and a pNPG transglycosidation product was found to accumulate through the duration of the experiment. In contrast, both the Y305C and Y305G substituted variants completely consumed the substrate within 3 h. A transient transglycosidation species was detected, but glucose was the major product by 4 h and shows that the transglycosidation reaction was reduced but not completely eliminated.

BGL reactions with natural substrate maintain a dynamic equilibrium since reaction species can participate in either hydrolytic or transglycosidation reactions [7, 8]. TLC analysis of reactions using BGL1, Y305C, Y305F, and Y305G at 50 mM cellobiose showed that the transglycosidation reactions were present for all of the tested enzymes and is consistent with the presence of transglycosidation product in reactions using pNPG. Since kinetic experiments showed lower inhibition of hydrolytic activity in reactions using a natural substrate than reactions using a synthetic substrate, we suspect that TLC analysis under these conditions lacks the sensitivity to differentiate wild-type and mutant BGLs.

## Discussion

In this study we tested a library of mutant *A. niger* BGL1 proteins using a two-step functional screen and identified several improved enzymes. In order to better understand the mechanisms of improvement, kinetic experiments were performed using the wild-type enzyme and several of the evolved variants. The most important finding showed that a Y305C substitution dramatically changed the kinetic parameters for both synthetic and natural substrates, reducing the inhibitory effect of high substrate concentrations towards the hydrolytic reaction. To our knowledge, these are the first experiments establishing the functional significance of the amino acid at position 305 in GH3 BGLs. BGLs have been extensively studied and inhibition by glucose and high substrate concentrations are well documented. These two characteristics limit the use of BGLs in industrial applications since high substrate loadings and the complete conversion of substrate to glucose are desirable.

Inhibition of the hydrolytic reaction at high substrate concentrations is evident in kinetic plots with non-saturating profiles [7, 8, 12, 36, 64]. We observed decreasing hydrolytic rates at substrate concentrations above *K*_*m*_ for BGL1 and the evolved mutants, with the exception of Y305C. This kinetic profile is consistent with previous reports and is caused by a competing transglycosidation reaction in which substrate also acts as an acceptor in the +1 subsite [8, 64]. Since the hydrolysis reaction of the Y305C variant lacked inhibition at high substrate concentrations, we investigated position 305 in several homologs and found that Phe was the most prevalent amino acid, while Tyr was only present in AnBGL1 and a homolog from *Rhizopus oryzae*. We used site-directed mutagenesis of the *bgl1* gene to test all of the residues identified by multiple sequence alignments and found that other substitutions with small functional groups (Gly, Ala, and Val) caused a similar loss of inhibition as the Y305C substitution. The Phe-substituted variant had a nearly identical kinetic profile as the wild-type Tyr, although the Phe^305^ variant was slightly less inhibited at high substrate concentrations than wild-type (BGL1 *K*_*i* pNPG_ = 2.98 ± 0.46 mM; Y305F: *K*_*i* pNPG_ = 4.78 ± 0.44 mM). It is likely that the GH3 BGLs with either Cyc, Gly, Val, or Ala at position 305 have similar kinetic profiles as the Y305C-substituted BGL1, and supports the hypothesis that transglycosidation is functionally significant in natural cellulase systems [64].

To investigate the structure/function relationship between position 305 residue and the transglycosidation reaction, we mapped its location using the crystal structure of a GH3 BGL from *A. aculeatus*. The AaBGL1 structure shows that three residues form the GH3 BGL +1 subsite and includes the position 305 residue. Since transglycosidation reactions are based on the affinity for acceptor in the +1 subsite, our data showing the control of hydrolytic versus transglycosidase activity by the position 305 residue is consistent with the AaBGL1 crystal structure. The substrate-binding pocket of GH3 BGLs is formed by highly conserved residues in the −1 and +1 subsites, though the distal subsites are less well conserved. Substituting Trp^68^ in BGL1 has also demonstrated the potential to reduce transglycosidase activity [36]. Unlike substitutions at position 305, those at position 68 cause a decrease in reaction rates for the hydrolysis reaction at substrate concentrations close to 2 × *K*_*m*_. Hydrolysis rates for mutants with substitutions at Trp^68^ are attenuated and reach saturation below the inhibited rates of the wild-type enzyme, and *k*_*cat*_ values are approximately fourfold lower than the wild-type enzyme [36]. In contrast, the position 305 substituted variants reached saturation above the wild-type _*app*_*V*_*max*_. Alignments show that the position 305 is the least well conserved residue in the active site. The position 305 residue might therefore acts as a “molecular tuning dial” between the hydrolysis and transglycosidase activities. A comparative study of six BGLS with either Phe (five homologs) or Tyr (one homolog) at position 305 showed that hydrolytic/transglycosidase activities can vary significantly between BGLs with very similar active sites [7]. While only small differences in *K*_*m*__hydrolysis_ values were observed (1.1–2.95 mM cellobiose), the range of *K*_*m* transglycosidation_ values was much greater (1.3–35 mM cellobiose). A relationship between *K*_*m hydrolysis*_ and *K*_*m transglycosidation*_ was established in kinetic profiles, whereby homologs with *K*_*m transglycosidation*_ approaching *K*_*m hydrolysis*_ were more inhibited in the production of glucose than those with *K*_*m transglycosidation*_ well above *K*_*m hydrolysis*_. Closer inspection of the amino acid sequences of these six BGLs and other homologs with either Phe^305^ or Tyr^305^ shows that a variable sequence forms part of the loop that coordinates this residue (Fig. 3c). It is possible that this variable sequence creates subtle changes in the orientation of the position 305 residue and could provide the basis for finer tuning between hydrolytic and transglycosidation reactions.

A second beneficial substitution, Q140L, increased hydrolytic activity by 156 % compared to BGL1 at 2 mM pNPG. The double-substituted Q140L/Y305C variant had slightly higher *K*_*m*_ and *K*_*i* glucose_ values compared to the Y305C background, suggesting decreased affinity for substrate and product in the active site. Since *K*_*i* glucose_ values could not be calculated for either wild-type or the Q140L BGL1 variant using a substrate inhibition model, we also fit the reaction rates for a substrate range of 0.1–1 mM pNPG to the Micheals–Menten equation under the competitive inhibition model allowing the *K*_*i* glucose_ for both enzymes to be approximated. This analysis showed an increase in *K*_*m*_ (BGL1 0.51 ± 0.04 mM pNPG; Q140L 0.74 ± 0.03 mM pNPG) that was consistent with the increase in *K*_*m*_ observed using data fit to the substrate inhibition model (BGL1 0.82 ± 0.12 mM pNPG; Q140L 1.09 ± 0.06 mM pNPG) and an increased *K*_*i* glucose_ value (BGL1 1.72 ± 0.09 mM; Q140L 2.73 ± 0.07 mM) for the Q140L variant compared to wild type. It is therefore possible that the *K*_*m transglycosidation*_ for the Q140L would be slightly higher than wild type, as we observed an increase in *K*_*i* pNPG_ (BGL1 2.98 ± 0.46 mM; Q140L 3.41 ± 0.21 mM). Even though Gln^140^ is not directly involved in forming the substrate-binding pocket, the proximity of the active site to the position 140 residue could explain the change in K_*m*_, *K*_*i* glucose_ and *K*_*i* pNPG_ values. In the AaBGL1 crystal structure and a Phyre^2^ homology model of BGL1 (data not shown), the side-chain nitrogen of Gln^140^ forms a hydrogen bond with backbone amide oxygen of Ser^93^. Since Asp^92^ forms part of the −1 subsite, it is possible that the loss of a hydrogen bond between Ser^93^ and Gln^140^ of Q140L would cause a subtle change in the affinity for cellobiose and glucose in the substrate-binding pocket, as well as a slight reduction in affinity for acceptor in the +1 subsite.

Three beneficial substitutions (A480V, K494Q, and N557D) were located in the β/α domain that mediates a protein–protein interaction between subunits of the AaBGL1 functional dimer. When tested alone or in combination with Y305C, no significant changes were observed in the kinetic constants for these mutants. Interestingly, the GH3 BGL from *A. fumigatus* has two of the three β/α domain substitutions naturally (Val^480^ and Asp^557^), and the GH3 BGL from *N. crassa* has one of the substitutions naturally (Val^480^). Because we did not determine *k*_*cat*_ values, the functional relationship between these substitutions and the observed increases in apparent *V*_*max*_ remains unclear. However, we suspect that the interaction between subunits could modulate activity though changes in the catalytic turnover number if the quaternary structure is conserved in GH3 BGLs. Alternatively, the β/α domain could be involved in subtle intramolecular domain interactions that lead to the increased apparent *V*_*max*_ caused by the A480V, K494Q, and N557 substitutions.

We also found that the signal peptide was altered in several evolved proteins, providing evidence that the MFα pre-sequence is not optimal for the heterologous expression and secretion of BGL1 in *S. cerevisiae*. Secretion of heterologously expressed proteins in yeast is often engineered using a signal peptide from the host organism [65–70]. In a previous study by our group, the expression of several fungal BGLs was tested in *S. cerevisiae* using the MFα signal peptide to mediate protein secretion [25]. While the BGLs had very similar amino acid sequences (>80 % identity) and multiple sequence alignments predict nearly identical substrate-binding pockets, activity varied significantly between enzymes. In the present study, we used a similar system such that the experimental wild-type protein was a fusion construct replacing the native BGL1 signal peptide with the MFα pre-sequence. Since the entire open reading frame was subjected to mutagenesis, we hypothesized that mutations in the MFα pre-sequence could increase expression or secretion. Six of the evolved BGLs were found to have mutations encoding modified signal sequences. We found that the residues at positions 21 or 22 of the MFα peptide were substituted in all of the evolved proteins with mutated signal sequences, where Val^22^ was replaced with either Ala or Asp in four of the twelve evolved proteins. In a directed evolution study using the MFα prepro-peptide to mediate ssFc secretion from *S. cerevisiae*, Rakestraw et al. [71] found a V22A substitution in all of the evolved proteins. Surprisingly, the V22A substitution had no effect when tested alone, and only produced an increase in secretion when combined with secondary mutations in the region encoding the MFα pro-sequence. In our study, we chose not to use the MFα pro-sequence since it has been shown to have negative effects on heterologous expression and secretion in a foreign host. [69] We identified two beneficial substitutions at position 22 of the MFα pre-sequence including the previously reported V22A substitution. These results show that a portion of the BGL1 polypeptide may function as a secretory pro-sequence and requires an optimized MFα pre-sequence for efficient secretion.

## Conclusions

In this study we applied directed evolution to *A. niger* BGL1 by expressing a library mutant BGLs in *S. cerevisiae* and used a two-step functional screen to identify improved enzymes. We found a key substitution at Tyr^305^ that dramatically reduced the inhibitory effect towards the hydrolytic activity of BGL1 that is caused by transglycosidation reactions at high substrate concentrations. Using the Y305C variant as a background allowed kinetic data to be fit to the Michaelis–Menten equation. This improved the characterization of Q140L, A480V, K494Q, and N557D variants and allowed kinetic constants to be determined in the absence of substrate inhibition. Experiments using the quadruple-substituted BGL1 variant, DLCV, also showed that the Y305C substituted enzyme can be used in combination with several beneficial substitutions, demonstrating a cumulative effect towards increased activity. This work provides a tool to better understand functional mechanisms that differentiate GH3 BGLs and shows that cellulases can be optimized for high substrate loadings, where the position 305 residue will likely be a critical component of BGL1 derivatives engineered for industrial applications.

## Methods

### Molecular biology

Experiments in yeast were performed using *S. cerevisiae* CEN.PK110-10C (*MAT*α *his3* Δ*1 MAL2*-*8C SUC2*). Yeast cultures were grown at 30 °C in YPD or yeast nitrogen base (YNB) supplemented as required to maintain the auxotrophic selection marker. Plasmids were constructed by in vivo homologous DNA recombination using a pGREG503 derivative with a unique KpnI site and the *HIS3* auxotrophic marker [72]. The plasmids were assembled into *S. cerevisiae* by co-transforming linearized plasmid and DNA fragments using the lithium acetate/carrier DNA method. DNA parts were designed with at least 50 bp regions of homology to mediate recombination. Transformants were cultured on solid media for selection (YNB + 2 % glucose containing 1.5 % agar and supplemented with synthetic dropout media without histidine). After transformation, assembled plasmids were extracted from *S. cerevisiae* and propagated in *E. coli* for sequencing. Verified constructs were transformed back into *S. cerevisiae* for subsequent experiments. DNA fragments used in assembling plasmids were amplified by PCR using Phusion High-Fidelity DNA polymerase (Thermo Scientific) and primers listed in Additional file 1: Table S1. For site-directed mutagenesis, full-length genes were constructed by PCR overlap extension using DNA parts amplified with primers containing the desired nucleotide substitutions. The DNA parts used for in vivo recombination were purified by gel extraction with the GeneJET Gel Extraction Kit (Thermo Scientific). Plasmids were purified from *E. coli* and *S. cerevisiae* using the GeneJET plasmid miniprep kit (Thermo Scientific). Yeast cells were treated with lytic enzyme (MP Biomedicals) for 1 h prior to the lysis step for plasmid extractions.

### Expression of a secreted BGL in *S. cerevisiae*

Three plasmids were constructed to express *bgl1* and assemble mutagenized gene libraries in *S. cerevisiae* (Fig. 1a). The assembly plasmid, pKL022, was constructed by co-transforming AscI/KpnI-linearized pGREG503, the *ScP*_TDH3_ promoter, and the *ScT*_CYC1_ terminator into *S. cerevisiae.* The promoter and terminator were amplified by PCR using pGREG503-based constructs as templates such that the 5′ end of the *ScP*_TDH3_ promoter and the 3′ end of the *ScT*_CYC1_ terminator had at least a 50 bp of homology with the linearized plasmid. The region between the *ScP*_TDH3_ promoter and the *ScT*_CYC1_ terminator was designed with an 86 nucleotide sequence that included a XhoI restriction site, linker, His_6_ tag, and a stop codon. A second plasmid, pKL024, was constructed similarly using the XhoI-linearized pKL022 and a 500-bp synthetic DNA fragment (Integrated DNA Technologies) designed to insert nucleotides encoding the MFα pre-sequence in pKL022. The BGL1 expression plasmid, pKL029, was constructed by co-transforming *S. cerevisiae* with XhoI-linearized pKL024 and the PCR-amplified *bgl1* gene [25]. Secretion was engineered to be under control of the *S. cerevisiae* MF*α* pre-peptide by replacing the first 21 codons from the wild-type gene with the first 24 codons from the *MFα* gene to construct *MFα*–*bgl1* fusion gene (experimental wild type). The primers used to amplify *bgl1* were designed to replace the native signal sequence with the MFα sequence at the 5′ end of the gene, and the linker region of the pKL022 and pKL024 plasmids at the 3′ end (Fig. 1a). PCR amplification of the *bgl1* gene from pKL029 with the primer pair KL50 and KL51 produced a DNA fragment that would recombine with XhoI-linearized pKL022 when co-transformed in *S. cerevisiae*, and would express a functionally secreted BGL.

### Error-prone PCR of *bgl1*

Error-prone PCR was used to generate sequence diversity of the *bgl1* gene. The region between the start codon and the linker region of pKL029 was mutagenized by PCR using Taq polymerase under high MgCl_2_ conditions (5 mM), with an unbalanced dNTP mixture (0.2 mM dATP, 0.2 mM dGTP, 1 mM dTTP, and 1 mM dCTP) and in the presence of MnCl_2_ (0.15 mM) using the KL50/KL51 primer pair. To generate a library of *bgl1* mutants, the PCR product from several reactions was pooled, mixed with XhoI-linearized pKL022 and transformed into *S. cerevisiae* by electroporation. Electrocompetent *S. cerevisiae* cells were prepared from 250 ml of a YPD culture (O.D._600_ = 1.6) in 50 ml of a 1 M sorbitol solution containing 0.1 M LiAc and 10 mM DTT incubated for 1 h at room temperature with gentle mixing. After the LiAc/DTT treatment, cells were washed twice in 50 ml of 1 M sorbitol and suspended to a final volume of 5 ml in ice-cold 1 M sorbitol. Aliquots (0.2 ml) of the electrocompetent cell suspension were removed, mixed with 1.5 μg of PCR product, 1.5 μg of XhoI-linearized pKL022, and electroporated (2.5 kV, 25 μF) using an ice-chilled cuvette. Cells from ten transformations were pooled, and a small quantity of the mixture was removed to determine the transformation efficiency. The remainder of the mixture was transferred to 100 ml of selective media and grown for 2 days at 30 °C with shaking. The transformation pool was then harvested by centrifugation, suspended in 10 mL of YNB containing 20 % glycerol and stored at −80 °C in 0.5 mL aliquots.

### Selection of improved BGLs

Improved BGLs were identified using a growth selection with cellobiose as the sole carbon source followed by an endpoint activity assay using *p*-nitrophenyl-β-D-glucopyranoside (pNPG) as a substrate. For growth selection, an overnight culture was used to inoculate 50 ml of fresh media containing glucose at an O.D._600_ = 0.05 and after 6 h cells were spread on solid YNB + 1 % cellobiose. The cell density for growth on solid media containing cellobiose was optimized to approximately 13 cells/cm^2^. Cells were also grown on solid YNB + 2 % glucose for comparison. After 3–4 days of growth, the largest colonies were picked and used to inoculate 200 μl of YNB + 2 % glucose in 96-well plates to supply enzymes for the endpoint activity assay. Four wells from each 96-well plate were inoculated with cells containing pKL029 to express BGL1 as a control. The 96-well plates were sealed in plastic bags and incubated at 30 °C without shaking for two days. Twenty μl of the culture was transferred to 180 μl of fresh media and grown for an additional 2 days at 30 °C without shaking. The O.D._600_ was measured for each culture and 50 μl of supernatant was transferred to 150 μl of a 66.6 mM citrate buffer, pH 5.0, containing 2.66 mM pNPG in 96-well plates. Reactions were incubated at room temperature for 30 min and quenched with 20 μl of 1 M NaOH. The amount of *p*-nitrophenol (pNP) released was determined by measuring the absorbance at 405 nm using the extinction coefficient 18 mM^−1^cm^−1^ and normalized to cell density. Strains with BGL activities greater than WT + 2SD were streaked on solid YNB + 2 % glucose media, and three colonies were re-tested using the same endpoint assay. Strains producing BGLs that exceeded a WT + 2SD threshold of activity were chosen for further analysis.

### BGL activity assays

The activities of BGL variants were compared to the experimental wild type using a time course assay measuring the release of pNP from pNPG. For protein production, fresh YNB + 2 % glucose media supplemented with synthetic dropout media lacking histidine was inoculated with 0.5 ml of overnight culture and grown at 30 °C for 48 h. Cells were removed by centrifugation and the supernatants were filtered using a 0.2 μM nylon membrane. Reactions were performed at 30 °C in 2 ml microcentrifuge tubes by adding 200 μl of 16 mM pNPG to a mixture of 1 ml of 80 mM citrate buffer, pH 5.0, and 400 μl of culture supernatant. Two hundred μl samples were removed from the reactions at time intervals over a ten-minute period (five data points) and transferred to 20 μl of 1 M NaOH. The amount of pNP released was measured at 405 nm and activities were calculated using the linear portion of the curve containing at least three time points. Reactions were performed in biological triplicate using cultures inoculated from individual colonies. Assay components were pre-incubated at 30 °C for 60 min prior to adding substrate.

### Enzyme kinetics

Kinetic experiments using pNPG as the substrate were performed in 96-well plates at 30 °C. Reactions were initiated by adding 50 μl of culture supernatant or 50 μl of a 0.01 μg/μl concentrated protein preparation to 150 μl of a 66 mM citrate buffer solution (pH 5.0) containing pNPG in a range from 0.1–10 mM (final concentration). Glucose was tested in a range from 0–100 mM (final concentration) at each pNPG concentration. Reactions were stopped 5 min after adding enzyme by transferring 100 μl of the assay mixture to 10 μl of 1 M NaOH in 96-well plates.

Assays using cellobiose as the substrate were performed in 96-well plates at 30 °C with a final reaction volume of 200 μl in 5 mM citrate buffer, pH 5.0. Cellobiose was tested at a range from 2–75 mM (final concentration). BGL1 was also tested at a 0.5 and 1 mM cellobiose (final concentration). Reactions were started by adding 50 μl of culture supernatant to 150 μl of the citrate/cellobiose solution and stopped after 5 min by transferring 100 μl of the mixture to 400 μl of glucose assay solution (62.5 mM Tris–HCL, pH 8.3, 1.25 mM ATP, 1.875 mM NAD, 12.5 mM MgCl_2_, 12.5 U/ml hexokinase (Sigma), and 12.5 U/ml glucose-6-phosphate dehydrogenase (Sigma)) and incubated for 30 min at room temperature. The amount of glucose released was determined by measuring the amount of NADH produced at 340 nm, using the extinction coefficient 6220 M^−1^cm^−1^.

For experiments using concentrated protein preparations, 100 ml of culture supernatant was filtered using a 10 kDa cutoff membrane (VivaSpin) to a final volume of 1 ml. The retentate was diluted to 15 ml using 5 mM citrate buffer, pH 5.0, and buffer exchange process was repeated twice before reducing the sample volume to 500–800 μl. Protein concentrations were determined using a Coomassie Protein Assay Kit (Thermo Scientific) with BSA as a standard. Concentrate samples were analyzed by SDS-PAGE with and without PNGase F (New England BioLabs) treatment (data not shown). Kinetic parameters for steady-state reactions were determined using GraphPad Prism enzyme kinetics module.

### Analysis of reaction products

Reactions using 40 mM pNPG and 50 mM cellobiose as substrate were performed using the same conditions as for enzyme kinetics. Ten μl samples were removed from 200 μl reactions at time intervals and stopped using 1 μl of 1 M NaOH. One μl aliquots from each time interval were applied to a silica TLC plates (Whatman), eluted with a *n*-butanol, ethyl acetate, 2-propanol, acetic acid, and water (1:3:2:1:1) and developed as previously described [63].

## Additional file

10.1186/s13068-016-0470-9 Primers used in this study.

## Abbreviations

BGL: β-glucosidase
CBP: consolidated bioprocessing
CBH: cellobiohydrolase
EGL: endoglucanase
GH3: glycosyl hydrolase family 3
MFα: *Saccharomyces cerevisiae* mating pheromone α factor
PCR: polyclonal chain reaction
pNPG: *p*-nitrophenyl-β-D-glucopyranoside
pNP: *p*-nitrophenol
TLC: thin-layer chromatography
