# Supplementary material for: Directed evolution of a fungal β-glucosidase in Saccharomyces cerevisiae
Source: Biotechnol Biofuels. 2016 Mar 3;9:52. doi: 10.1186/s13068-016-0470-9 (PMC4778352; doi:10.1186/s13068-016-0470-9)
Supplement: Supplementary file 1 — 10.1186/s13068-016-0470-9 Primers used in this study. [file 13068_2016_470_MOESM1_ESM.docx]

**Table S1.** Primers used in this study.

| Primer | Sequence | Description |
| --- | --- | --- |
| C1:506 | TAACCCTCACTAAAGGGAACAAAAGCTGGAGCTCGTTTAAACGGCGCGCCGAGACTGCAGCATTACTTTGAGAAG | Forward primer with homology to pGREG vectors and 5' end of *ScP_TDH3_*. |
| GC22 | CGATACTAACGCCGCCATCC | Reverse primer with homology to pGREG vectors. Used to amplify *ScT_CYC1_*. |
| KL46 | gtcttttttttagttttaaaacaccaagaacttagtttcgaaaaacaatgttggcctactccccgccgtattac | Forward primer with homology to *A. niger bgl1* gene. Removes natural signal sequence. Used for recombination into pKL022. |
| KL47 | gttcttctcctttactcatgaattcgccagaaccagcagcggagccagcggatccgtgaacagtaggcagagacgcc | Reverse primer with homology to *A. niger bgl1* gene. Used for recombination into pKL022. Removes stop codon and adds a homology sequence to linker region of the assembly sequence. |
| KL50 | gtcttttttttagttttaaaacaccaagaacttagtttcgaaaaacaatg | Forward primer to with homology to 3' region of *ScP_TDH3_* in pKL022, pKL024, and pKL029. |
| KL51 | gttcttctcctttactcatgaattcgccagaaccagcagcggagccagcggatcc | Reverse primer to with homology to the linker region in the assembly sequence of pKL022 and pKL024 and 3’ end of *bgl1* encoded in pKL029. |
| KL67 | actgcagttttattcgcagcatcctccgcattagctgctccagtcaacactttggcctactccccgccgtattac | Used with KL51 to add 50 bp encoding MFα pre sequence to *A. niger bgl1* gene using pKL012 as a template to construct pKL029 from pKL024. |
| KL93 | tctggcgaattcatgagtaaaggagaagaacttcatcatcatcatcatcattaatcatgtaattagttatgtcacgc | Forward primer used to construct pKL022. Use with GC22. Other PCR reaction uses C1:506 and KL51 pair. |
| KL107 | CAGCATCCTCCGCATTAGCTGCTaCAGTCAACACTTTGGCCTACTCC | Forward primer to generate 61C>A mutation. Encodes P21T. |
| KL108 | CAGCATCCTCCGCATTAGCTGCTtCAGTCAACACTTTGGCCTACTCC | Forward primer to generate 61C>T mutation. Encodes P21S. |
| KL109 | CATCCTCCGCATTAGCTGCTCCAGcCAACACTTTGGCCTACTCCCCGCCG | Forward primer to generate 65T>C mutation. Encodes V22A. |
| KL110 | CATCCTCCGCATTAGCTGCTCCAGaCAACACTTTGGCCTACTCCCCGCCG | Forward primer to generate 65T>A mutation. Encodes V22D. |
| KL112 | GTGACAAGGGTGCTGATATCCtATTGGGTCCAGCTGCCGGCCCTC | Forward primer to generate 428A>T mutation. Encodes Q140L. |
| KL113 | GCGCGAACCTCGACGATAAGACcATGCATGAGCTGTACCTCTGGCC | Forward primer to generate 681T>C mutation. Silent. |
| KL114 | GGACATGTCTATGCCGGGAGACGcCGATTACGACAGTGGCACGTC | Forward primer to generate 917T>C mutation. Encodes V303A. |
| KL115 | GTCTATGCCGGGAGACGTCGATTgCGACAGTGGCACGTCTTACTGGG | Forward primer to generate 923A>G mutation. Encodes Y305C. |
| KL116 | CGTGCAACGCAACCATAGCGAGcTGATCCGCCGTATTGGAGCAGAC | Forward primer to generate 1180T>C mutation. Silent. |
| KL117 | GAACAAGAATGGCGTATTCACTGtGACCGATAACTGGGCTATTGATC | Forward primer to generate 1448C>T mutation. Encodes A480V. |
| KL118 | GATCAGATTGAGGCGCTTGCTcAGACCGCCAGTGTCTCTCTTGTC | Forward primer to generate 1489A>C mutation. Encodes K494Q. |
| KL119 | CTTGCTAAGACCGCCAGTGTCTCaCTTGTCTTTGTCAACGCCGACTC | Forward primer to generate 1506T>A mutation. Silent. |
| KL120 | GAGGGTTATATCAATGTCGACGGtAACCTGGGTGACCGCAGGAACC | Forward primer to generate 1557A>T mutation. Silent. |
| KL122 | CTCTGTCGGCCCAGTCTTGGTTgACGAGTGGTACGACAACCCCAATG | Forward primer to generate 1678A>G mutation. Encodes N557D. |
| KL123 | GGTACGACAACCCCAATGTTACtGCTATTCTCTGGGGTGGTCTTC | Forward primer to generate 1707C>T mutation. Silent. |
| KL126 | CGCCCTTCACCTGGGGCAAGACcCGTGAGGCCTACCAAGATTAC | Forward primer to generate 1818T>C mutation. Silent. |
| KL127 | CATTGACTACCGCGGATTTGACAtGCGCAACGAGACTCCTATCTATG | Forward primer to generate 1925A>T mutation. Encodes K639M. |
| KL128 | CCGCGGATTTGACAAGCGCAACGtGACTCCTATCTATGAGTTCGGC | Forward primer to generate 1934A>T mutation. Encodes Q642V. |
| KL129 | CTGAGGCAGCGCCGACTTTCGGtGAGGTCGGAAATGCGTCGGATTAC | Forward primer to generate 2067A>T mutation. Silent. |
| KL130 | GCGTCGGATTACCTCTACCCCGAaGGACTGCAGAGAATCACCAAGTTC | Forward primer to generate 2103T>A mutation. Silent. |
| KL131 | GGCAAGGTTGCGGGTGATGAAGTaCCTCAACTGTATGTTTCTCTTGG | Forward primer to generate 2349T>A mutation. Silent. |
| KL133 | GGAGTAGGCCAAAGTGTTGACTGtAGCAGCTAATGCGGAGGATGCTG | Reverse primer to generate 61C>A mutation. Encodes P21T. |
| KL134 | GGAGTAGGCCAAAGTGTTGACTGaAGCAGCTAATGCGGAGGATGCTG | Reverse primer to generate 61C>T mutation. Encodes P21S. |
| KL135 | CGGCGGGGAGTAGGCCAAAGTGTTGgCTGGAGCAGCTAATGCGGAGGATG | Reverse primer to generate 65T>C mutation. Encodes V22A. |
| KL136 | CGGCGGGGAGTAGGCCAAAGTGTTGtCTGGAGCAGCTAATGCGGAGGATG | Reverse primer to generate 65T>A mutation. Encodes V22D. |
| KL138 | GAGGGCCGGCAGCTGGACCCAATaGGATATCAGCACCCTTGTCAC | Reverse primer to generate 428A>T mutation. Encodes Q140L. |
| KL139 | GGCCAGAGGTACAGCTCATGCATgGTCTTATCGTCGAGGTTCGCGC | Reverse primer to generate 681T>C mutation. Silent. |
| KL140 | GACGTGCCACTGTCGTAATCGgCGTCTCCCGGCATAGACATGTCC | Reverse primer to generate 917T>C mutation. Encodes V303A. |
| KL141 | CCCAGTAAGACGTGCCACTGTCGcAATCGACGTCTCCCGGCATAGAC | Reverse primer to generate 923A>G mutation. Encodes Y305C. |
| KL142 | GTCTGCTCCAATACGGCGGATCAgCTCGCTATGGTTGCGTTGCACG | Reverse primer to generate 1180T>C mutation. Silent. |
| KL143 | GATCAATAGCCCAGTTATCGGTCaCAGTGAATACGCCATTCTTGTTC | Reverse primer to generate 1448C>T mutation. Encodes A480V. |
| KL144 | GACAAGAGAGACACTGGCGGTCTgAGCAAGCGCCTCAATCTGATC | Reverse primer to generate 1489A>C mutation. Encodes K494Q. |
| KL145 | GAGTCGGCGTTGACAAAGACAAGtGAGACACTGGCGGTCTTAGCAAG | Reverse primer to generate 1506T>A mutation. Silent. |
| KL146 | GGTTCCTGCGGTCACCCAGGTTaCCGTCGACATTGATATAACCCTC | Reverse primer to generate 1557A>T mutation. Silent. |
| KL148 | CATTGGGGTTGTCGTACCACTCGTcAACCAAGACTGGGCCGACAGAG | Reverse primer to generate 1678A>G mutation. Encodes N557D. |
| KL149 | GAAGACCACCCCAGAGAATAGCaGTAACATTGGGGTTGTCGTACC | Reverse primer to generate 1707C>T mutation. Silent. |
| KL152 | GTAATCTTGGTAGGCCTCACGgGTCTTGCCCCAGGTGAAGGGCG | Reverse primer to generate 1818T>C mutation. Silent. |
| KL153 | CATAGATAGGAGTCTCGTTGCGCaTGTCAAATCCGCGGTAGTCAATG | Reverse primer to generate 1925A>T mutation. Encodes K639M. |
| KL154 | GCCGAACTCATAGATAGGAGTCaCGTTGCGCTTGTCAAATCCGCGG | Reverse primer to generate 1934A>T mutation. Encodes Q642V. |
| KL155 | GTAATCCGACGCATTTCCGACCTCaCCGAAAGTCGGCGCTGCCTCAG | Reverse primer to generate 2067A>T mutation. Silent. |
| KL156 | GAACTTGGTGATTCTCTGCAGTCCtTCGGGGTAGAGGTAATCCGACGC | Reverse primer to generate 2103T>A mutation. Silent. |
| KL157 | CCAAGAGAAACATACAGTTGAGGtACTTCATCACCCGCAACCTTGCC | Reverse primer to generate 2349T>A mutation. Silent. |
| KL173 | GTCTATGCCGGGAGACGTCGATTtCGACAGTGGCACGTCTTACTGGG | Forward primer used to generate Y305F. |
| KL174 | CCCAGTAAGACGTGCCACTGTCGaAATCGACGTCTCCCGGCATAGAC | Reverse primer used to generate Y305F. |
| KL175 | GTCTATGCCGGGAGACGTCGATTggGACAGTGGCACGTCTTACTGGG | Forward primer used to generate Y305W. |
| KL176 | CCCAGTAAGACGTGCCACTGTCccAATCGACGTCTCCCGGCATAGAC | Reverse primer used to generate Y305W. |
| KL177 | GTCTATGCCGGGAGACGTCGATggCGACAGTGGCACGTCTTACTGGG | Forward primer used to generate Y305G. |
| KL178 | CCCAGTAAGACGTGCCACTGTCGccATCGACGTCTCCCGGCATAGAC | Reverse primer used to generate Y305G. |
| KL179 | GTCTATGCCGGGAGACGTCGATgtCGACAGTGGCACGTCTTACTGGG | Forward primer used to generate Y305V. |
| KL180 | CCCAGTAAGACGTGCCACTGTCGacATCGACGTCTCCCGGCATAGAC | Reverse primer used to generate Y305V. |
| KL181 | GTCTATGCCGGGAGACGTCGATgcCGACAGTGGCACGTCTTACTGGG | Forward primer used to generate Y305A. |
| KL182 | CCCAGTAAGACGTGCCACTGTCGgcATCGACGTCTCCCGGCATAGAC | Reverse primer used to generate Y305A. |

*Mutations are written in lower case.
